# Multiple Impacts of Loss of Plastidic Phosphatidylglycerol Biosynthesis on Photosynthesis during Seedling Growth of Arabidopsis

**DOI:** 10.3389/fpls.2016.00336

**Published:** 2016-03-21

**Authors:** Koichi Kobayashi, Kaichiro Endo, Hajime Wada

**Affiliations:** ^1^Department of Life Sciences, Graduate School of Arts and Sciences, The University of TokyoTokyo, Japan; ^2^Core Research for Evolutional Science and Technology, Japan Science and Technology AgencyTokyo, Japan

**Keywords:** phosphatidylglycerol, photosynthesis, thylakoid membrane lipid, chloroplast, photosystem I, photosystem II, electron transport, photodamage

## Abstract

Phosphatidylglycerol (PG) is the only major phospholipid in the thylakoid membrane in cyanobacteria and plant chloroplasts. Although PG accounts only for ~10% of total thylakoid lipids, it plays indispensable roles in oxygenic photosynthesis. In contrast to the comprehensive analyses of PG-deprived mutants in cyanobacteria, *in vivo* roles of PG in photosynthesis during plant growth remain elusive. In this study, we characterized the photosynthesis of an *Arabidopsis thaliana* T-DNA insertional mutant (*pgp1-2*), which lacks plastidic PG biosynthesis. In the *pgp1-2* mutant, energy transfer from antenna pigments to the photosystem II (PSII) reaction center was severely impaired, which resulted in low photochemical efficiency of PSII. Unlike in the wild type, in *pgp1-2*, the PSII complexes were susceptible to photodamage by red light irradiation. Manganese ions were mostly dissociated from protein systems in *pgp1-2*, with oxygen-evolving activity of PSII absent in the mutant thylakoids. The oxygen-evolving complex may be disrupted in *pgp1-2*, which may accelerate the photodamage to PSII by red light. On the acceptor side of the mutant PSII, decreased electron-accepting capacity was observed along with impaired electron transfer. Although the reaction center of PSI was relatively active in *pgp1-2* compared to the severe impairment in PSII, the cyclic electron transport was dysfunctional. Chlorophyll fluorescence analysis at 77K revealed that PG may not be needed for the self-organization of the macromolecular protein network in grana thylakoids but is essential for the assembly of antenna-reaction center complexes. Our data clearly show that thylakoid glycolipids cannot substitute for the role of PG in photosynthesis during plant growth.

## Introduction

The thylakoid lipid bilayer formed by amphipathic glycerolipids serves as a matrix embedded with photosynthesis protein–cofactor complexes forming the electron transport chain. The lipid composition of the thylakoid membrane is highly conserved among cyanobacteria and plant chloroplasts, with glycolipids as major constituents (Mizusawa and Wada, 2012). The only major phospholipid in the thylakoid membrane is phosphatidylglycerol (PG), an anionic lipid with a negative charge in the phosphoglyceryl head group attached to the diacylglycerol backbone. Although PG accounts only for ~10 mol% of total thylakoid lipids, this lipid has crucial roles in oxygenic photosynthesis, as described later.

In addition to having a role as building blocks of the thylakoid membrane, lipids serve as structural components of membrane protein complexes to support their structure and function. Many lipid molecules are present in photosystem I (PSI) and PSII complexes. Crystallography analysis of the PSII dimer complex from *Thermosynechococcus vulcanus* at 1.9-Å resolution identified 20 lipid molecules per monomer in the structure; 5 molecules were PG, which were buried near the reaction center with their head groups facing the cytoplasmic side (Umena et al., 2011). In the PSII complex, 3 PGs were located around the primary electron acceptor (Q_A_) binding site, one was present at the interface between D1 and CP43 and the other was located near the secondary electron acceptor (Q_B_) binding site. In the crystal structure of the PSI complex from *Thermosynechococcus elongatus* at 2.5-Å resolution, 4 lipid molecules were assigned per monomer, and 3 of the 4 molecules were PG (Jordan et al., 2001). One of the 3 PG molecules was located near the reaction center, and the other 2 located between the PsaB and PsaX subunits and near the monomer–monomer interface of the trimeric PSI complex, respectively. Moreover, x-ray crystallography analysis of the light-harvesting complex I (LHCI)-PSI complex from pea at 2.8 Å identified 6 PG molecules in addition to 4 molecules of other thylakoid lipids; 3 PGs were found in the PSI core and another 3 were in the LHCI complex (Qin et al., 2015). PG is also structurally involved in LHCII; in LHCII crystal structures from spinach at 2.7 Å (Liu et al., 2004) and pea at 2.5 Å (Standfuss et al., 2005), one PG molecule was buried at the monomer-monomer interface with the *trans*-Δ^3^-hexadecenoic acid at the *sn*-2 position penetrating the deep binding pocket of the trimer.

The role of PG in photosynthesis was initially examined in phospholipase-treated thylakoid membranes in which PG was specifically degraded. Elimination of up to 70% of the original PG from pea thylakoid membranes disrupted electron transport in PSII without decreasing PSI activity (Jordan et al., 1983; Droppa et al., 1995). Moreover, the PSII dimer from spinach (Kruse et al., 2000) and LHCII trimers from pea (Nussberger et al., 1993) were dissociated into monomers by phospholipase A_2_ treatment, which suggests a requirement of PG for the structural organization of the PSII-LHCII complex. Likewise, phospholipase A_2_ treatment to thylakoid membranes from *Arabidopsis thaliana* inhibited the PSII electron transport at both donor and acceptor sides and disassembled the PSII–LHCII complexes into PSII and LHCII monomers (Kim et al., 2007).

Reverse genetic approaches have greatly contributed to unraveling the functions of PG in oxygenic photosynthesis. Both in plants and cyanobacteria, PG is synthesized from phosphatidic acid through PG phosphate (PGP) as an intermediate (Mizusawa and Wada, 2012). In *Synechocystis* sp. PCC 6803 (hereafter *Synechocystis* 6803), a mutant of the *pgsA* gene encoding PGP synthase was made and characterized to study the role of PG (Hagio et al., 2000). The mutant was unable to synthesize PG but could grow photoautotrophically with PG exogenously supplemented to the growth media. After deprivation of PG from the growth media, the photosynthetic activity of the *pgsA* mutant rapidly decreased with concomitant decrease of PG content, mainly due to the severely impaired PSII function. In the mutant, deprivation of PG inhibited electron transport from Q_A_ to Q_B_ (Gombos et al., 2002; Itoh et al., 2012) and destabilized the Mn cluster of the oxygen-evolving complex by dissociating extrinsic proteins (PsbO, PsbV, and PsbU) from the PSII core (Sakurai et al., 2007). The activity of PSI was decreased in *pgsA* as well but only after a longer period of PG deficiency (Domonkos et al., 2004). Long-term PG deprivation induced accumulation of PSI monomer with a decrease in PSI trimer, which is consistent with the existence of a PG molecule in the monomer-monomer interface of PSI from *T. elongatus* (Jordan et al., 2001).

In higher plants, PG is synthesized in plastids, mitochondria, and endoplasmic reticula (ER) membranes (Wada and Murata, 2007). In *A. thaliana*, two isoforms of PGP synthase (PGP1 and PGP2) have been identified; PGP1 is dual localized to plastids and mitochondria (Babiychuk et al., 2003), whereas PGP2 is in the ER (Tanoue et al., 2014). Knockout mutations of PGP1 decreased PG content in leaves by ~80% from wild-type levels and impaired thylakoid membrane development in chloroplasts (Hagio et al., 2002; Babiychuk et al., 2003). In a T-DNA insertional PGP1 knockout mutant (*pgp1-2*), amounts of PSI and PSII core proteins were substantially decreased with strong transcriptional downregulation of photosynthesis-associated genes (Kobayashi et al., 2015). The photochemical efficiency of PSII was greatly decreased in *pgp1-2*, and further depletion of PG in the mutant by phosphate starvation caused complete loss of the PSII activity (Kobayashi et al., 2015). Despite the severe perturbation of chloroplast functions in *pgp1-2*, the morphology of mitochondria was similar to that in the wild type (Hagio et al., 2002; Babiychuk et al., 2003). A double knockout mutant of PGP1 and PGP2 (*pgp1-2 pgp2*) has an embryonic lethal phenotype with complete loss of PG biosynthesizing activity (Tanoue et al., 2014), so PG biosynthesis by PGP2 at the ER can complement the loss of PGP1 activity in mitochondria but not in chloroplasts in the *pgp1-2* mutant.

Studies in phospholipase-treated thylakoid membranes of plants and in cyanobacterial mutants deficient in PG biosynthesis have elucidated a special requirement of PG for photochemical and electron transport reactions particularly in PSII, as described earlier. Meanwhile, some parts of the PG functions can be substituted by sulfoquinovosyldiacylglycerol (SQDG), another anionic lipid in the thylakoid membrane (Güler et al., 1996; Essigmann et al., 1998; Yu and Benning, 2003). Moreover, as suggested by no or negligible photosynthetic defects in a PGP1 point mutant (*pgp1-1*) with an 80% reduction in the PGP1 activity (Xu et al., 2002; Yu and Benning, 2003), the effect of reduced PG content on photosynthesis may be alleviated to some extent by fine-tuning the photosynthetic systems and lipid metabolism during plant growth. By contrast, the effect of severe loss of plastidic PG biosynthesis on photosynthesis during plant growth is largely unknown.

To gain deep insight into *in vivo* effects of PG biosynthesis on photosynthetic machinery during plant growth, we examined the activity and functionality of photosynthetic components in the PGP1-knockout *pgp1-2* mutant.

## Materials and methods

### Plant materials and growth conditions

The wild type and *pgp1-2* mutant (the KG10062 line; Hagio et al., 2002) were the Columbia ecotype of *A. thaliana*. Surface-sterilized seeds were cold-treated at 4°C for 3 days in the dark before germination. Unless otherwise stated, plants were grown on Murashige and Skoog medium (adjusted to pH 5.7 with KOH) containing 3% (w/v) sucrose solidified with 0.8% (w/v) agar in plates at 23°C under continuous white light (15 μmol photon m^−2^ s^−1^). Wild-type and *pgp1-2* plants were grown in a growth chamber (CLE-303, Tomy Seiko, Tokyo) for 14 and 21 days, respectively, to equalize their developmental stages.

### Pigment determination

Lipophilic pigments were extracted with 80% (v/v) acetone from leaves or thylakoid membrane fractions, and debris was removed by centrifugation at 10,000 × *g* for 5 min. The absorbance of the supernatant at 720, 663.2, 646.8, 645, and 470 nm was measured by using the V-730 BIO spectrophotometer (JASCO, Tokyo). The chlorophyll (chl *a* and *b*) and carotenoid contents were determined as described in Melis et al. (1987) and Lichtenthaler (1987), respectively.

### Imaging analysis of chlorophyll fluorescence

Seedlings grown on agar plates were dark-incubated for 15 min before measurement. Minimum chlorophyll fluorescence (Fo) and maximum quantum yield of PSII (Fv/Fm) were determined under a saturating pulse with the IMAGING-PAM MAXI chlorophyll fluorometer and the ImagingWin software (Heinz Walz, Effeltrich, Germany). Lowest intensity of measuring light was used to minimize the photosynthetic effect of measuring light.

### Photochemical efficiency analysis under light

Photochemical efficiency of PSII under actinic light in leaves was determined with the JUNIOR-PAM chlorophyll fluorometer and the WinControl-3 software (Heinz Walz). Light-response curves of effective quantum yield of PSII (Y_II_) and quantum yield of non-regulated energy dissipation (Y_NO_) were determined as described in Kobayashi et al. (2013b). To determine the contribution of photoinhibition-related non-photochemical quenching (qI) to total non-photochemical quenching (qN), leaves dark-adapted for 15 min were exposed to light stress (125 μmol photon m^−2^ s^−1^) for 10 min and total qN was measured at the end of the light stress. After relaxation of the rapidly reversible qN component with additional dark treatment for 15 min, the remaining qN component was determined as qI.

### Measurement of red-light induced photoinhibition

Leaves detached from seedlings were vacuum-infiltrated with 150 mM sorbitol containing 0 or 1 mg/ml lincomycin and incubated under dim light at room temperature for 3 h. Then leaves were illuminated with or without red LED light (660 nm peak wavelength) (VBL-SL150-RB, Valore, Kyoto, Japan) for 1 h. Red light intensity was measured with the LI-250A light meter and the LI-190SA quantum sensor (LI-COR, Lincoln, USA). Fv/Fm in illuminated leaves was measured with the JUNIOR-PAM fluorometer under lowest measuring light intensity after incubation in darkness for 15 min.

### Electron paramagnetic resonance (EPR) spectroscopy

Seedlings were incubated in a growth chamber at 23 or 45°C for 16 h before measurement. EPR spectra from Mn^2+^ were directly obtained from fresh leaves placed in a quartz EPR tube (Tokyo Chemical Industry, Tokyo) with a JES-TE300 X-band (9.2 GHz) spectrometer (JEOL, Tokyo) at room temperature. Measurement conditions were frequency = ~9.18 GHz, power = ~1.0 mW, center field = 325 mT, sweep width = 50 mT, sweep time = 4 min, modulation width = 2 mT, amplitude = 500–1000, time constant = 0.3 s. Signal intensity was normalized with fresh weight of leaf samples.

### Measurement of fast-induction kinetics of chlorophyll fluorescence in leaves

Leaves were treated with or without 40 μM 3-(3,4-dichlorophenyl)-1,1-dimethylurea (DCMU) in 150 mM sorbitol by vacuum infiltration, followed by dark-incubation for 5 min. Chlorophyll fluorescence transients in leaves were measured in a logarithmic time series between 30 μs and 20 s after the onset of strong actinic light (1650 μmol photon m^−2^ s^−1^) with a light-emitting diode pump-probe spectrometer (JTS-10, BioLogic, Claix, France).

### Measurement of decay kinetics of chlorophyll fluorescence in thylakoid membrane fractions

Thylakoid membrane fractions of 5 μg/ml chlorophyll concentration were prepared under dim light as described in Allahverdiyeva et al. (2007), but with skipping the washing step with a low osmotic buffer. When required, 40 μM DCMU or 400 μM 1,4-benzoquinone (BQ) was added to the thylakoid fraction. We confirmed that 0.1 % (v/v) ethanol and 1% (v/v) dimethyl sulfoxide, the solvents used to dissolve DCMU and BQ, respectively, did not affect the kinetics. Decay kinetics of chlorophyll fluorescence following a single saturation flash was measured between 0.2 ms and 60 s with the FL3500 fluorometer (Photon Systems Instruments, Brno, Czech). Because small artificial signals were recorded even in the blank sample containing only water, they are subtracted from data as background noises. Fv/Fm values of thylakoid samples were calculated from the minimum (Fo) and maximum (Fm) yields of chlorophyll fluorescence measured before and just after the saturating excitation, respectively.

### Chlorophyll fluorescence analysis in thylakoid membranes at 77K

To prepare stacked thylakoid fractions, seedlings were homogenized in a cold grinding buffer (0.4 M sucrose, 5 mM MgCl_2_, 10 mM NaCl, 2 mM EDTA, 2 g/L bovine serum albumin, 2 g/L ascorbic acid, HEPES-NaOH, pH 7.5) under dim light. The homogenates were filtered through a single layer of Miracloth (Merck Millipore, Darmstadt, Germany). After centrifugation at 6000 × *g* for 4 min at 4°C, the pellet was resuspended in a cold resuspension buffer (0.4 M sucrose, 5 mM MgCl_2_, 10 mM NaCl, HEPES-NaOH, pH 7.5) to obtain 1 μg/ml chlorophyll-containing membrane fractions. To prepare unstacked thylakoids, MgCl_2_ and NaCl were eliminated from both the grinding buffer and resuspension buffer. A restacked membrane fraction was obtained by adding 5 mM MgCl_2_ and 10 mM NaCl in a final concentration to the unstacked thylakoid samples, followed by incubation for 1 h at 4°C.

Fluorescence emission spectra of chlorophyll proteins at 77K were obtained from thylakoid membrane fractions in liquid nitrogen by using the RF-5300PC spectrofluorometer (Shimadzu, Kyoto, Japan) under 435- nm excitation.

### Measurement of oxygen-evolving activity in thylakoid membrane fractions

Thylakoid membranes were prepared as described in Allahverdiyeva et al. (2007). Steady-state rate of oxygen evolution from thylakoids was measured as 23°C with a Clark-type oxygen electrode (Hansatech, King's Lynn, UK) using 1 mM 2, 6-dimethyl-1, 4-benzoquinone (DMBQ) under saturating white light.

### P700 absorbance measurement

The light-response curves corresponding to the different redox states of P700 were determined in a batch of leaves at room temperature by measuring the absorbance change at 830–875 nm as a reference with a Dual-PAM-100 (Heinz Walz). Maximum P700 change (Pm) was determined under a saturating far-red light irradiation for 20 s without saturation pulse flash, because saturation pulses caused small artificial signals unaffected by both DCMU and methylviologen (MV) treatments, which was significant in the case of the *pgp1-2* leaves emitting only low signals. The minimum P700 level (Po) in the absence of light was taken as the baseline and the steady-state P700 level (P) was recorded under actinic light. Using these values the ratio of oxidized P700^+^ to total P700 under a given light treatment was calculated as (P-Po)/(Pm-Po). When required, 40 μM DCMU was used to block electron transfer from PSII.

P700 oxidation-reduction kinetics in leaves was measured by absorbance changes at 705 nm under a weak far-red excitation with the JTS-10 spectrometer (BioLogic). When required, leaf samples were treated with 1 mM MV by vacuum infiltration before measurement to block cyclic electron transport around PSI.

## Results

### Impaired photosynthesis with decreased chlorophyll content in *pgp1-2*

**Figure 1:**
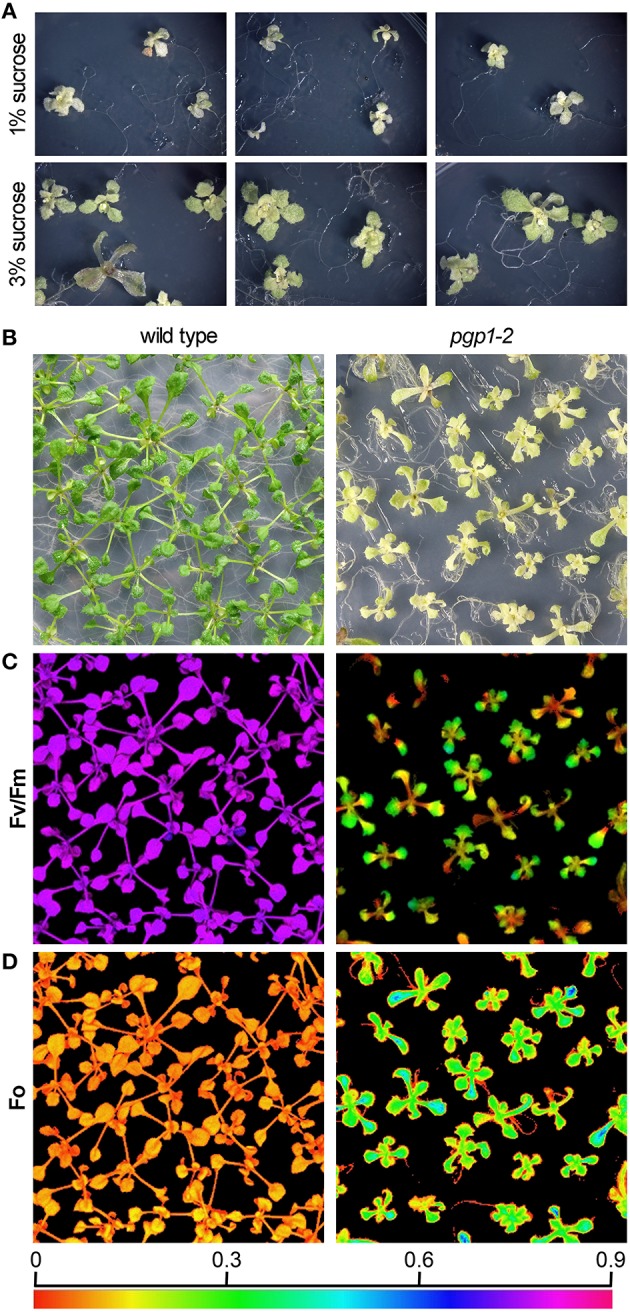
**Images of growth and photosynthesis of *pgp1-2* mutant plants in the presence of 3% sucrose**. **(A)** Growth phenotypes of *pgp1-2* seedlings grown for 21 days on agar media containing 1 or 3% sucrose. **(B)** Comparison of growth of 21-days-old *pgp1-2* seedlings with 14-days-old wild type. **(C,D)** Images of **(C)** maximum quantum yield (Fv/Fm) and **(D)** minimum chlorophyll fluorescence (Fo) in dark-adapted seedlings. Seedlings were the same as shown in **(B)** for both wild type and *pgp1-2* plants. The color in **(C,D)** represents the value of each parameter in the color scale.

The *pgp1-2* mutant requires exogenous sugars for growth (Hagio et al., 2002). This mutant showed better growth with supplementation of 3% (w/v) sucrose than with 1% sucrose in the growth medium (Figure 1A). Even in the presence of 3% sucrose, the growth of *pgp1-2* was slower than that of wild type; 21-days-old *pgp1-2* seedlings were at a growth stage similar to that of 14-days-old wild-type plants (Figure 1B). Decreasing light intensity from about 15 μmol photon m^−2^ s^−1^ to < 5 μmol photon m^−2^ s^−1^ did not alleviate the impaired leaf development, whereas stronger light intensity (~75 μmol photon m^−2^ s^−1^) damaged the *pgp1-2* seedlings (Supplemental Figure 1). Thus, we compared 21-days-old *pgp1-2* seedlings with 14-days-old wild-type seedlings under the 3% sucrose condition with 15 μmol photon m^−2^ s^−1^ continuous light for further experiments.

We previously reported that the *pgp1-2* mutation substantially decreased chlorophyll accumulation (Kobayashi et al., 2015). As represented by the pale yellow-green leaf color, chlorophyll content in the first and second true leaves of the *pgp1-2* mutant was only 3% of wild-type levels on a fresh weight basis even under the growth condition with 3% sucrose (Table 1). Carotenoid content was also substantially decreased in *pgp1-2* leaves. In the mutant, ratios of chl *a* to chl *b* and to carotenoids were decreased as compared with the wild type (Table 1), which suggests greater decrease of photosystem reaction centers than light-harvesting antennas in *pgp1-2*.

**Table 1:** **Pigment composition in the first and second leaves of seedlings**.

	**Chl *a* nmol g^−1^ FW**	**Chl *b* nmol g^−1^ FW**	**Carotenoids μg g^−1^ FW**	**Chl *a*/*b* mol/mol**	**Chl *a*/Carotenoids mmol/g**
Wild type	2276.6±117.1	734.2±37.0	463.8±27.0	3.10±0.01	4.91±0.22
*pgp1-2*	60.6±2.4	28.1±0.9	26.6±0.6	2.16±0.06	2.28±0.12

*Values are mean ± SD (n = 4). Chl, chlorophyll*.

Unlike in the wild type, in the *pgp1-2* mutant, chloroplast development in leaves is non-uniform and is limited around vascular tissues in leaves (Hagio et al., 2002; Kobayashi et al., 2015). To examine a spatial pattern of photosynthetic activity in the *pgp1-2* mutant, we determined Fv/Fm in whole seedlings using an imaging pulse amplitude modulation fluorometer (Figure 1C). Consistent with our previous analysis that *pgp1-2* plants had reduced Fv/Fm levels in leaves (Kobayashi et al., 2015), the mutant showed very low Fv/Fm values in whole seedlings, with particularly low signals around the shoot apical meristem and petioles. The low Fv/Fm was attributed to high Fo values representing large emission of chlorophyll fluorescence in the dark-adapted state (Figure 1D). The higher Fo with lower Fv/Fm in *pgp1-2* mutant than the wild type was also confirmed in thylakoid membrane samples by single-turnover flash-induced chlorophyll fluorescence measurement (Table 2). These data suggest that PG deficiency globally occurs in photosynthetic membranes and limits the photosynthetic electron transfer in the *pgp1-2* mutant.

**Table 2:** **PSII activity in isolated thylakoid membranes**.

	**Fo (a.u.)**	**Fm (a.u.)**	**Fv/Fm**	**O_2_ evolution**
Wild type	0.073 ± 0.009	0.266 ± 0.031	0.727 ± 0.006	190±16
*pgp1-2*	0.326 ± 0.031	0.385 ± 0.045	0.151 ± 0.024	22±4

*Thylakoid membrane samples corresponding to 5 μg chlorophyll ml^−1^ were used for both plants. Values are mean ± SD (n = 8 for fluorescence analysis and 3 for O_2_ evolution). Fo, minimum yield; Fm, maximum yield; Fv/Fm, maximum quantum yield of PSII. The steady-state rates of O_2_ evolution (μmol O_2_ mg chlorophyll^−1^ h^−1^) was measured in the presence of 1 mM DMBQ*.

### PG deficiency in *pgp1-2* plants increases the light susceptibility

**Figure 2:**
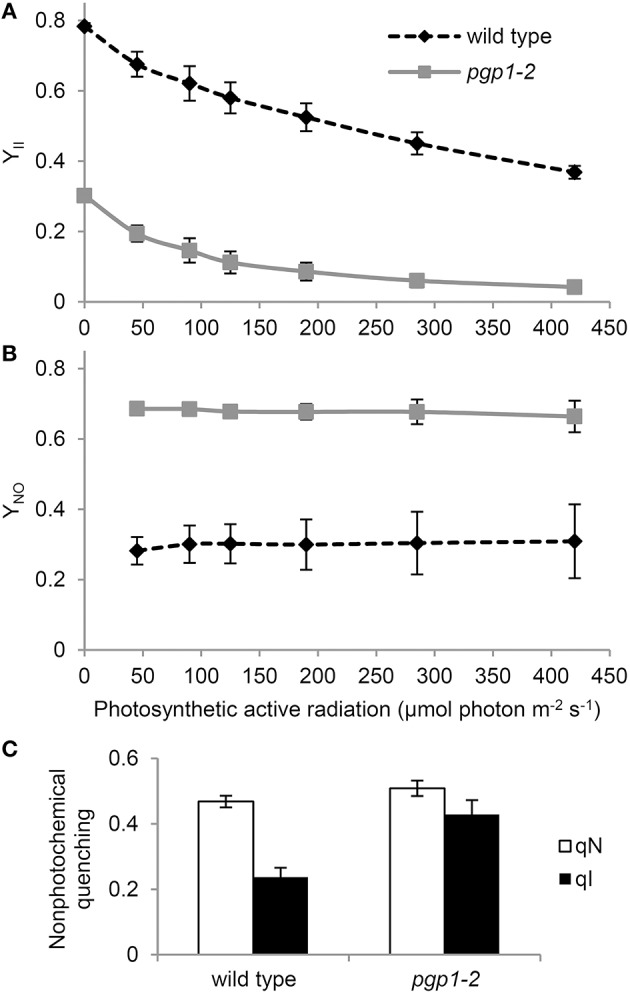
**Photosynthetic parameters of wild-type and *pgp1-2* seedlings**. **(A,B)** Light-response curves of **(A)** effective quantum yield of PSII (Y_II_) and **(B)** quantum yield of non-regulated energy dissipation (Y_NO_). **(C)** Contribution of photoinhibition (qI) to total non-photochemical quenching (qN) under 125 μmol photon m^−2^ s^−1^ photosynthetic active radiation. Data are mean ± SE from 3 independent experiments.

To further characterize the functionality of photosynthetic machinery in the *pgp1-2* mutant, we analyzed the quantum yield of PSII (Y_II_) and non-regulated energy dissipation (Y_NO_) under increased actinic light intensity. Both wild-type and *pgp1-2* leaves showed a gradual decrease in Y_II_ in response to increased light intensity (Figure 2A). The Y_II_ values were substantially lower in the *pgp1-2* mutant than the wild type mainly due to the decreased intrinsic photochemical efficiency of PSII represented by low Fv/Fm (Figure 1C). Meanwhile, Y_NO_ values were consistently higher in *pgp1-2* plants than the wild type under all ranges of light intensity (Figure 2B). The higher proportion of light energy dissipation in a non-regulated form (Y_NO_) implies loss of photoprotective mechanisms in the *pgp1-2* mutant (Kramer et al., 2004). Although the level of non-photochemical quenching (qN) was similar in the *pgp1-2* mutant and the wild type, the proportion of the photoinhibitory component (qI) to total qN was substantially higher in the mutant; in fact, most of the qN was attributed to the qI component in the mutant (Figure 2C).

**Figure 3:**
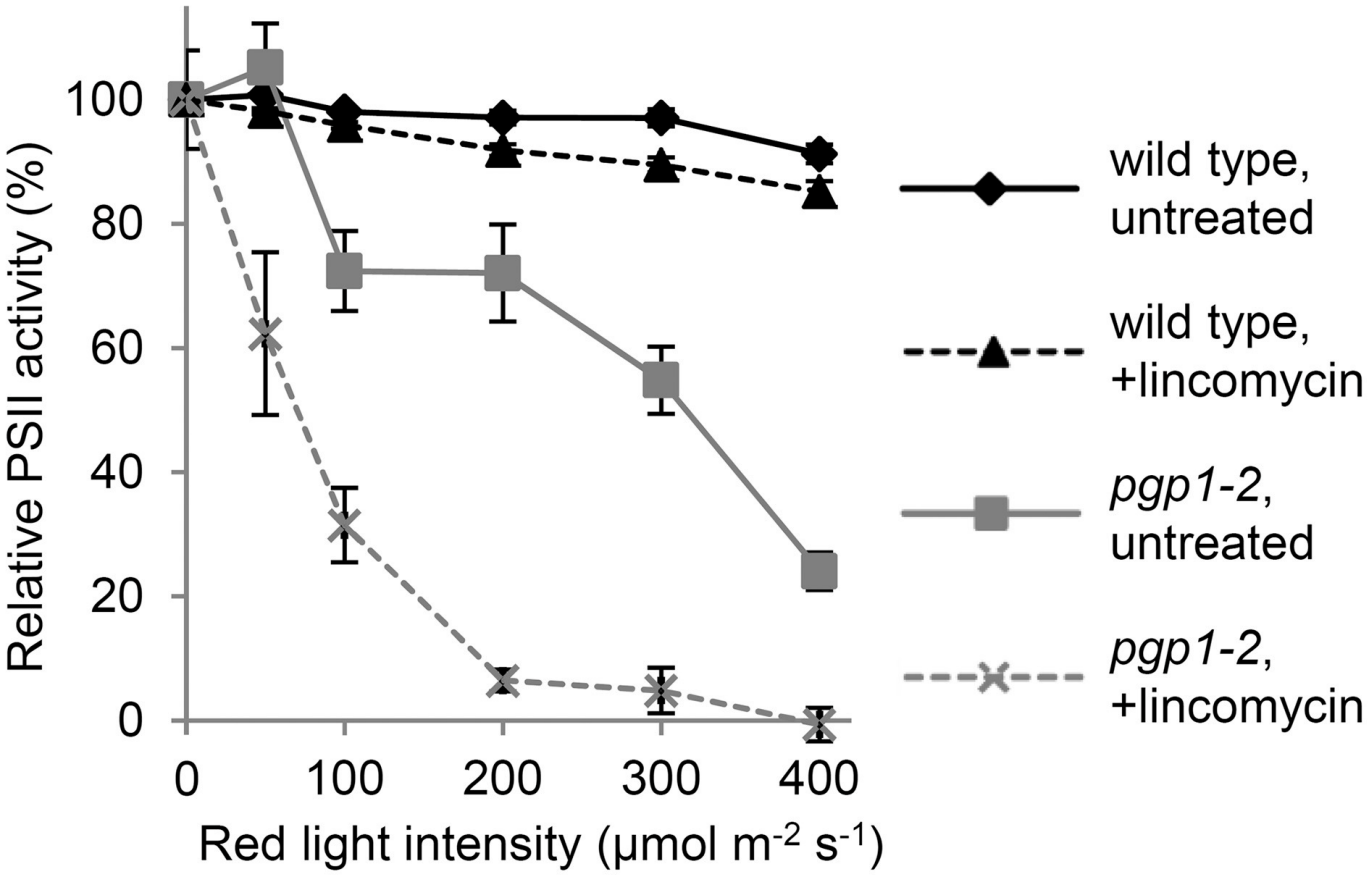
**Photoinhibition of Photosystem II (PSII) activity under red-light irradiation**. Maximum quantum yield of PSII (Fv/Fm) was determined as a measure of PSII activity after red light irradiation for 1 h. Before red light irradiation, leaves were treated with or without lincomycin for 3 h. Data are mean ± SE from 8 independent experiments.

To investigate the photoinhibition mechanism in *pgp1-2* plants, we illuminated leaves with various intensities of red light in the presence or absence of lincomycin, an inhibitor of protein synthesis in chloroplasts, for 1 h and then measured Fv/Fm values in the leaves after dark adaptation for 15 min (Figure 3). In wild-type leaves, Fv/Fm was only slightly decreased in response to increased red light intensities even in the presence of lincomycin. The data were consistent with observations that red light has little photoinhibitory effect on undamaged leaves (Ohnishi et al., 2005; Sarvikas et al., 2006; Takahashi et al., 2010). By contrast, in the absence of lincomycin, Fv/Fm was strongly decreased in *pgp1-2* leaves in response to increased red light intensity, and the decrease was further enhanced by lincomycin treatment. These data indicate that photoinhibition of PSII is strongly enhanced particularly in the damage process in *pgp1-2* mutant.

### Oxygen-evolving activity of PSII is abolished in *pgp1-2*

**Figure 4:**
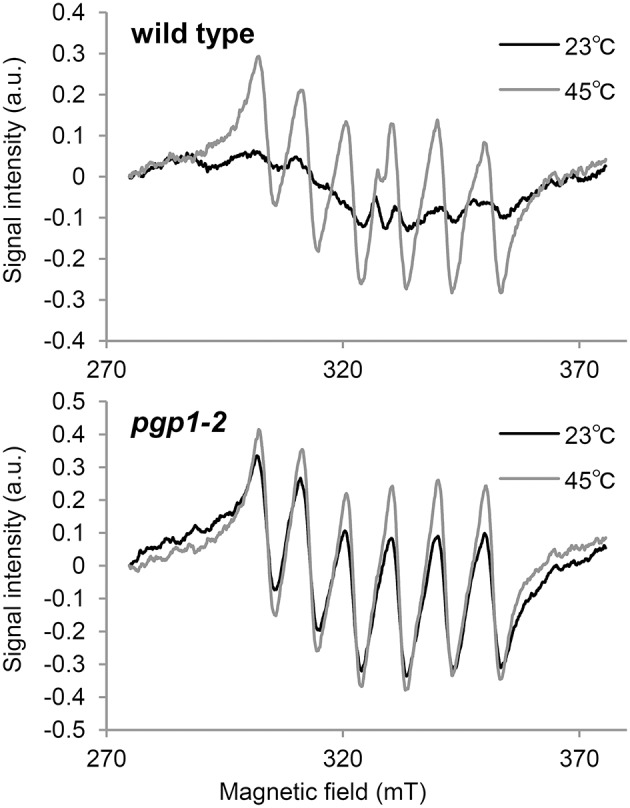
**Electron paramagnetic resonance spectra of Mn^2+^ in leaves incubated for 23 or 45°C for 16 h**. Signal intensity was normalized to fresh weight of the samples. Data are means from 3 independent experiments.

In the *Synechocystis* 6803 *pgsA* mutant, the Mn cluster of the oxygen-evolving complex is destabilized by PG deprivation with the extrinsic proteins being dissociated from the PSII complex (Sakurai et al., 2007). To examine the state of Mn in *pgp1-2* cells *in vivo*, we performed EPR spectroscopic analysis in intact leaves (Figure 4). The feature of the EPR-spectrum of protein-unbound Mn^2+^ is its sextet signals (Morsy and Khaled, 2002). In wild-type seedlings incubated at 23°C, no clear sextet lines from Mn^2+^ were detected. However, Mn^2+^ signals emerged with incubation at 45°C. Thus, in the wild type, most Mn^2+^ ions are integrated into protein systems but are dissociated from the complexes by heat treatment. In *pgp1-2* seedlings, fine sextet lines of Mn^2+^ were detected even at 23°C, so Mn^2+^ ions exist as a free form in the *pgp1-2* seedlings.

To directly determine the PSII activity in the *pgp1-2* mutant, oxygen-evolving activity was measured in the thylakoid membrane fraction in the presence of the artificial electron acceptor DMBQ (Table 2). Consistent with the result in the *Synechocystis* 6803 *pgsA* mutant, oxygen-evolving activity was almost abolished in the *pgp1-2* mutant.

### Energy transfer from antenna pigments to the PSII reaction center is severely impaired in *pgp1-2* mutant

**Figure 5:**
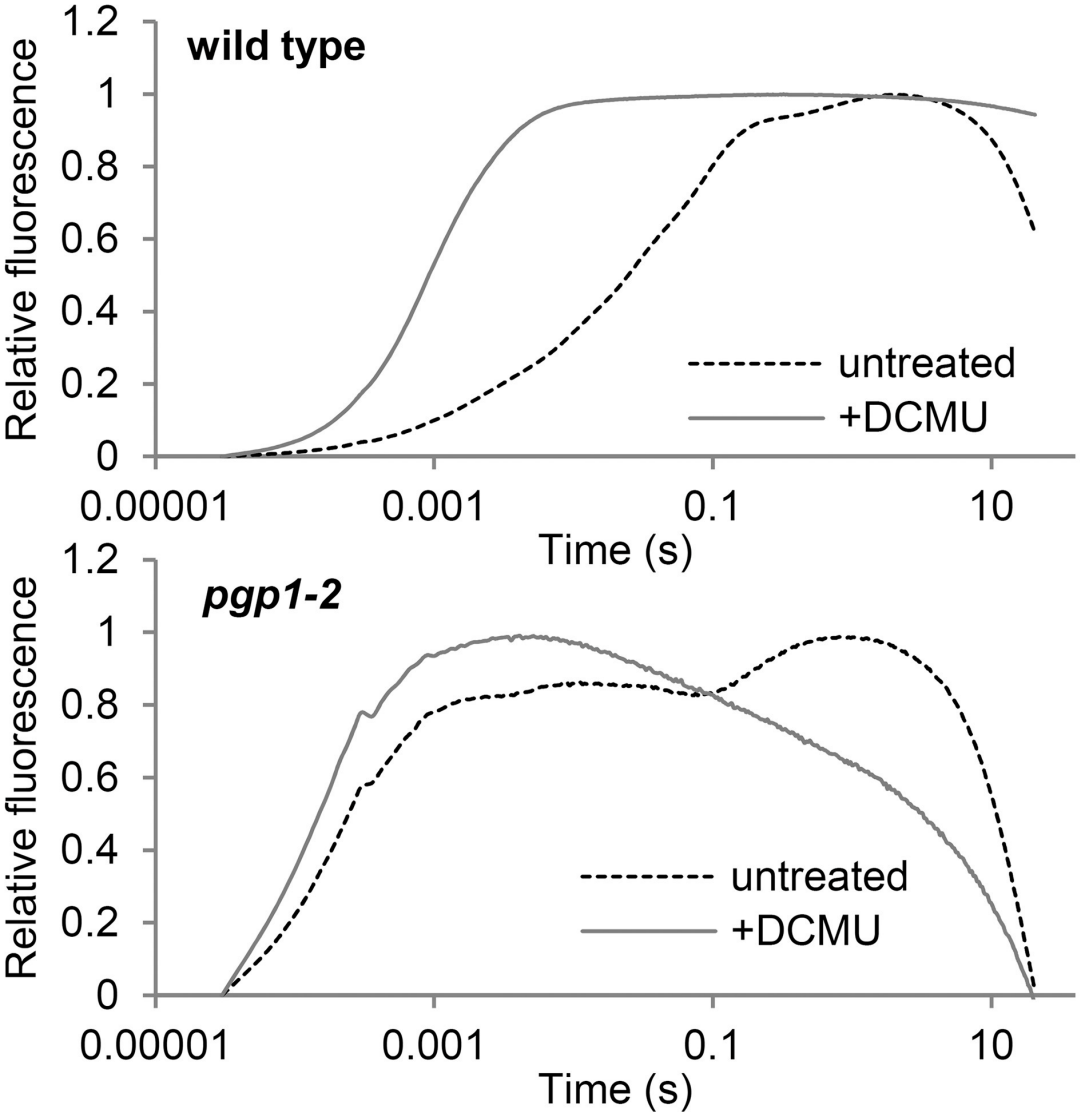
**Transient fluorescence induction kinetics of chlorophyll in leaves treated with or without DCMU**. Data are means from 3 independent experiments.

To evaluate electron transport activity in the *pgp1-2* mutant, we analyzed the induction kinetics of chlorophyll fluorescence in leaves in a logarithmic time series (Figure 5). The wild-type leaves showed a slow polyphasic increase of chlorophyll fluorescence after actinic light irradiation, which is explained by a stepwise retardation of the photosynthetic electron flow from primary photochemical reactions in PSII to later reduction processes at the acceptor side of PSI (Krause, 1991). In the presence of DCMU, which inhibits electron transport from Q_A_ to Q_B_, chlorophyll fluorescence rapidly peaked because reoxidation of Q${}_{\text{A}}^{-}$ was inhibited. In *pgp1-2* leaves, chlorophyll fluorescence quickly increased in the absence of DCMU. In the presence of DCMU, fluorescence increase was further accelerated, although the fluorescence decreased after peaking at about 5 ms. Very fast increase of chlorophyll fluorescence in the *pgp1-2* mutant suggests that the electron-transfer capability of PSII is very limited in the mutant. In *pgp1-2* leaves, particularly in the presence of DCMU, continuous irradiation of strong actinic light might severely damage PSII complexes and cause strong quenching of chlorophyll fluorescence.

### Different characteristics of electron transfer at the PSII acceptor side between *pgp1-2* and *synechocystis* 6803 *pgsA* mutant

**Figure 6:**
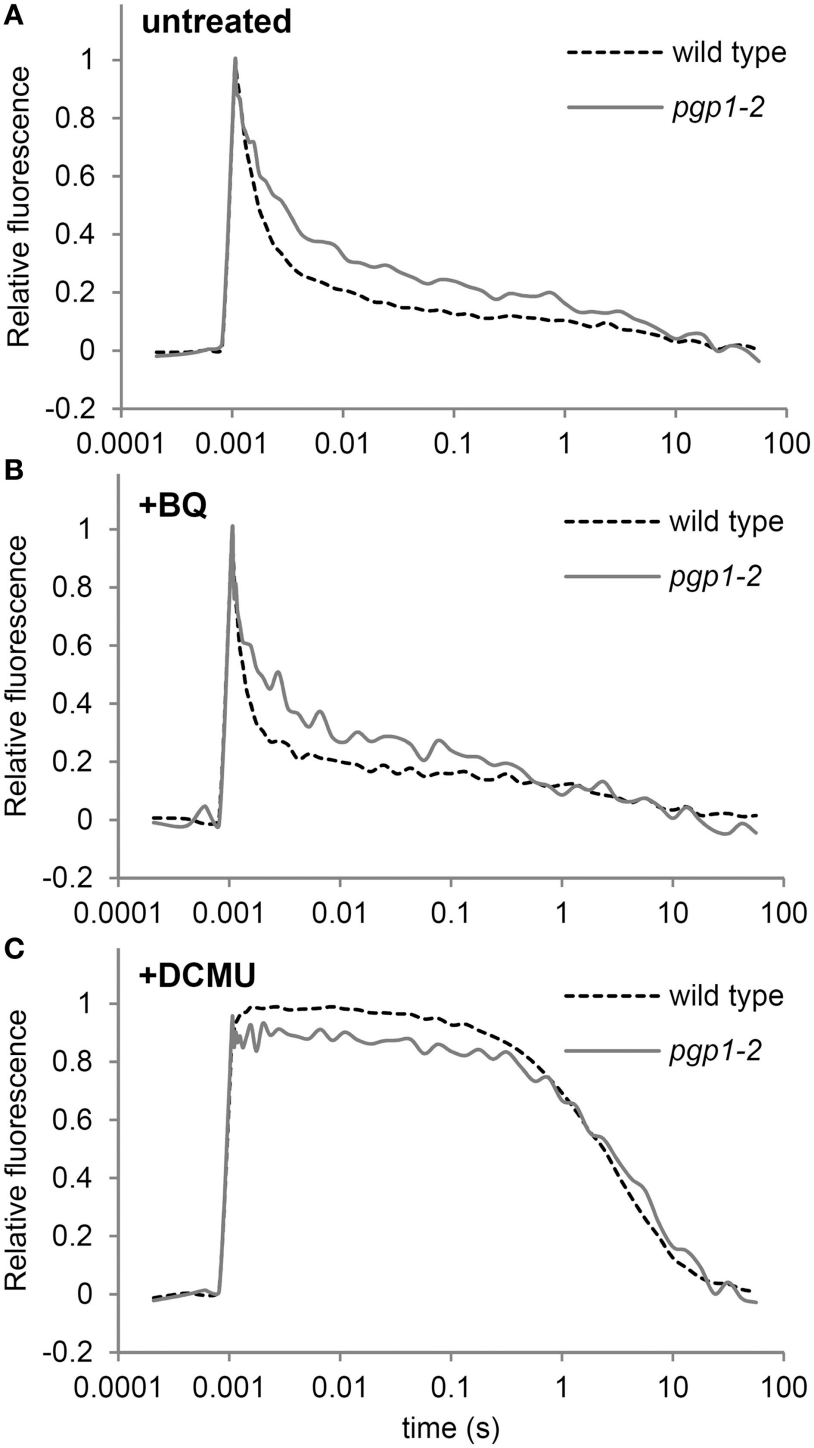
**Q${}_{\text{A}}^{-}$ reoxidation kinetics following a single saturating flash in thylakoid membrane fractions**. Fluorescence decay kinetics of chlorophyll were measured **(A)** in the absence or **(B)** presence of 1,4-benzoquinone (BQ) and **(C)** DCMU. Thylakoid membrane samples corresponding to 5 μg/ml chlorophyll were used for all measurements. Data are means from 4 independent experiments.

In the *Synechocystis* 6803 *pgsA* mutant, PG deprivation strongly impairs electron transfer from Q_A_ to Q_B_, particularly in the presence of artificial electron acceptors such as BQ (Hagio et al., 2000; Gombos et al., 2002; Itoh et al., 2012). To ascertain whether this also occurs in the *pgp1-2* mutant, we analyzed decay of single flash-induced chlorophyll fluorescence in thylakoid membrane fractions from wild-type and *pgp1-2* leaves (Figure 6). The decay profiles reflect reoxidation kinetics of Q_A_, which can be divided into 3 processes (Krause, 1991): (1) fast fluorescence decay (< 1 ms) related to the electron transfer from Q${}_{\text{A}}^{-}$ to a plastoquinone bound to the Q_B_ pocket; (2) middle exponential decay (~2 ms) related to the reoxidation of Q${}_{\text{A}}^{-}$ by a plastoquinone molecule moving from the plastoquinone pool to the empty Q_B_ pocket; and (3) slow decay (~1 s) related to reoxidation of Q${}_{\text{A}}^{-}$ by charge recombination with donor-side components.

In the absence of BQ, fluorescence decay was slower for the *pgp1-2* mutant than the wild type through the fast to the middle phase (Figure 6A), which suggests that reoxidation of Q${}_{\text{A}}^{-}$ by Q_B_ and the plastoquinone pool is impaired in *pgp1-2* thylakoids. In the wild type, BQ treatment accelerated the decay of chlorophyll fluorescence particularly in the middle phase (Figure 6B). In *pgp1-2* leaves, the fluorescence decay was almost unchanged by BQ treatment, which contrasted with the strong impairment of Q${}_{\text{A}}^{-}$ reoxidation with BQ supplementation in the *pgsA* mutant (Itoh et al., 2012). In Figure 6C, the variable fluorescence decays with similar kinetics in wild type and the *pgp1-2* mutant. The slow fluorescence decay in the DCMU-treated sample mainly resulted from charge recombination between Q${}_{\text{A}}^{-}$ and the donor-side components. These data suggest that charge recombination with the donor-side components is not notably affected in the mutant but reoxidation of Q${}_{\text{A}}^{-}$ at the acceptor side is retarded independently of the effect of artificial quinones.

### Cyclic electron flow around PSI is dysfunctional in the *pgp1-2* mutant

**Figure 7:**
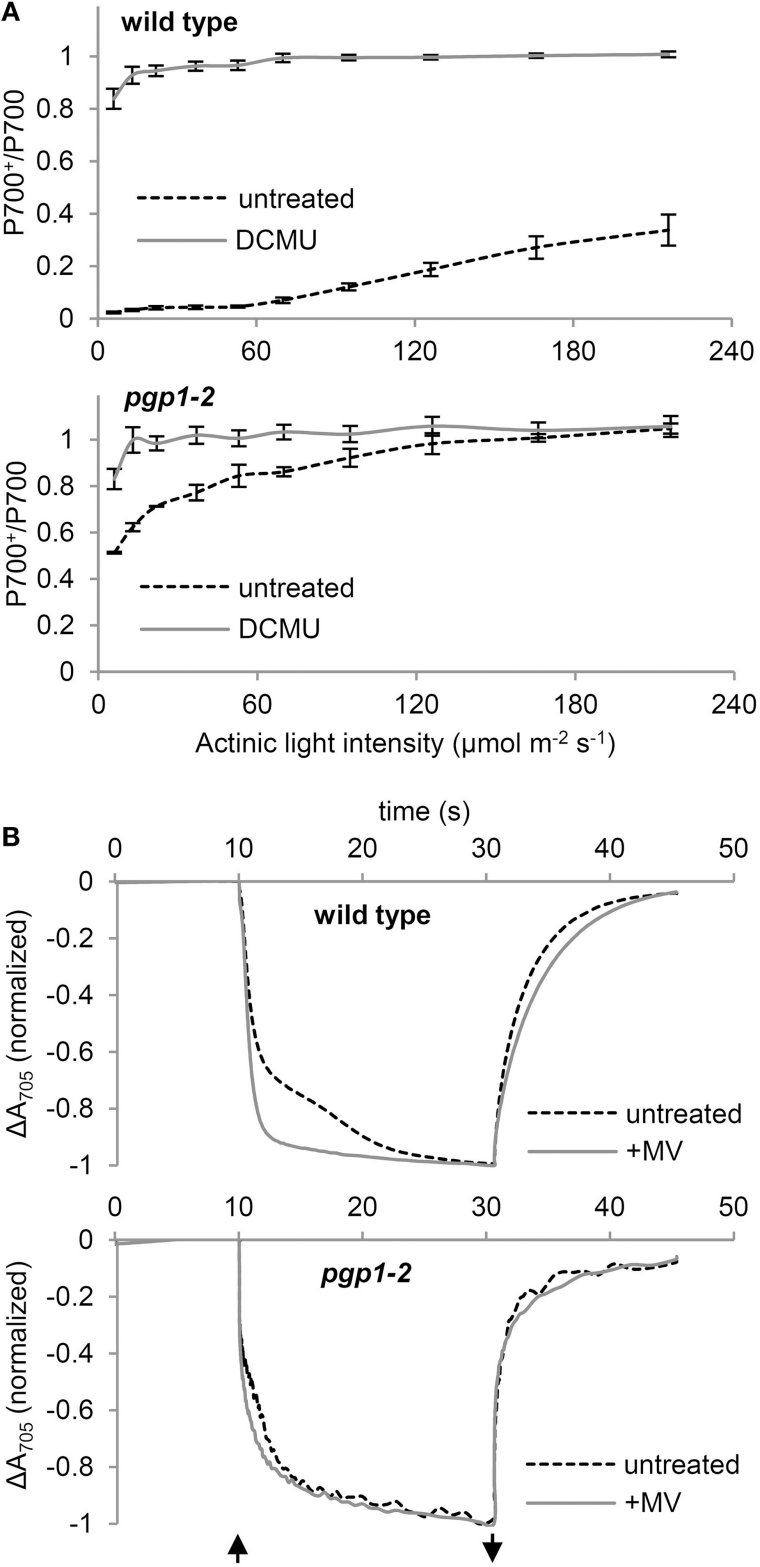
**P700 oxidation kinetics in leaf samples**. **(A)** Light-response curve of P700 oxidation in the absence or presence of DCMU. **(B)** P700 oxidation-reduction kinetics in response to far-red light irradiation in the absence or presence of 1 mM methylviologen (MV). The upward and downward arrows represent the start and end of far-red light illumination, respectively.

To assess whether the PSI activity is also affected by the *pgp1-2* mutation, we examined the redox state of the primary electron donor chlorophyll (P700) of the PSI reaction center under increased actinic light intensity (Figure 7A). In the presence of DCMU, P700 in both wild-type and *pgp1-2* leaves was mostly oxidized even under low actinic light conditions because of the blockage of electron transfer from PSII by DCMU. In the absence of DCMU, P700 in wild-type leaves was highly reduced under low actinic light mainly by linear electron transfer from PSII but was gradually oxidized in response to increased light intensity. By contrast, P700 in *pgp1-2* leaves was highly oxidized under low actinic light irradiation in the absence of DCMU. The data suggest that the electron donation from PSI to the acceptor side is more active than electron supply to PSI from the donor side.

To further assess the functionality of PSI in the *pgp1-2* mutant, kinetics of P700 oxidation by far-red light irradiation was measured in leaves by monitoring absorbance at 705 nm in the presence or absence of 1 mM MV (Figure 7B). MV with this high concentration efficiently accepts electrons from all PSI and thus abolishes the cyclic electron flow around PSI. In wild-type leaves treated with MV, rapid oxidation of P700 was observed as was reported previously (Joliot and Joliot, 2005). However, in the absence of MV, kinetics of P700 oxidation was slowed, which indicates reinjection of electrons to P700 via the cyclic pathway. In the *pgp1-2* leaves, P700 oxidation kinetics were similar with and without MV. The data suggest that cyclic electron flow is impaired in the *pgp1-2* mutant. Moreover, as compared with wild-type leaves treated with MV, P700 oxidation kinetics were slower in MV-treated *pgp1-2* leaves.

### PG deficiency in *pgp1-2* plants perturbs interaction between antenna complexes and reaction centers

**Figure 8:**
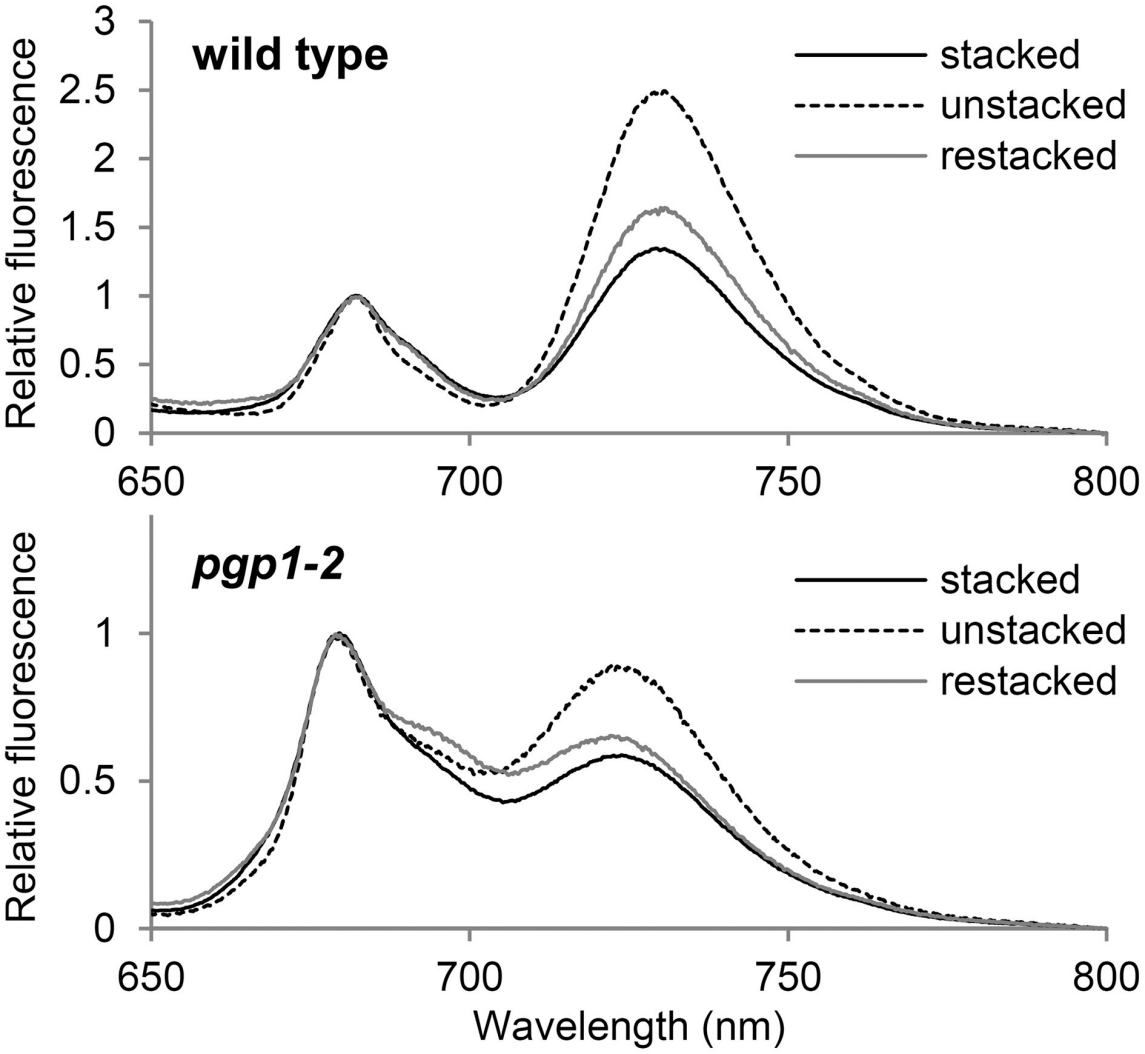
**Chlorophyll fluorescence spectra at 77K in the thylakoid membrane fraction (1 μg/ml chlorophyll)**. Stacked and unstacked samples were prepared with or without 5 mM MgCl_2_ and 10 mM NaCl, respectively. Restacked membranes were prepared by incubating unstacked thylakoid samples with 5 mM MgCl_2_ and 10 mM NaCl for 1 h.

Thylakoid membrane lipids greatly affect energetic interactions between reaction centers and antenna complexes. In fact, chlorophyll fluorescence at 77K, which reflects interaction states of photosystem–antenna complexes and structural organization of thylakoids (Krause, 1991; Andreeva et al., 2003; Kirchhoff et al., 2007), was remarkably changed in mutants defective in thylakoid lipid biosynthesis, including the *pgp1-2* mutant (Härtel et al., 1997; Kobayashi et al., 2013a, 2015). To gain insight into the effect of the lack of PG on protein arrangement in the thylakoid membrane, we examined the effect of cations on membrane organization in the *pgp1-2* mutant by measuring chlorophyll fluorescence spectra at 77K (Figure 8). The thylakoid membrane fraction from wild-type leaves showed two major peaks at 682 nm from the PSII-LHCII complex and at 731 nm from the PSI-LHCI complex. In the presence of cations (NaCl and MgCl_2_), LHCII was tightly connected with PSII in stacked grana regions (Kirchhoff et al., 2007), which resulted in high fluorescence emission from the PSII complexes (Figure 8). Depletion of cations in the buffer induced unstacking of the grana thylakoids and caused a spillover of excitation energy from LHCII to PSI as indicated by increased fluorescence emission from the PSI complex. Supplementation of cations to unstacked thylakoids induced restacking and decreased the emission from PSI probably due to a reduced energetic connection between PSI and LHCII (Kirchhoff et al., 2007).

As reported previously (Kobayashi et al., 2015), in *pgp1-2* thylakoids, emissions from PSII and PSI were both blue-shifted to 679 and 724 nm, respectively, which implies dissociation of antenna complexes from each reaction center. As observed in wild-type samples, fluorescence emission from PSI relative to that from PSII was increased in the *pgp1-2* mutant by preparing thylakoids in the absence of cations but was decreased with the addition of cations to the membrane samples (Figure 8). The changes in energy distribution between PSII and PSI did not accompany notable shifts of peak wavelength, which suggests that global rearrangement of the thylakoid membrane environment does not affect an intrinsic structure of photosystem core-antenna complexes in the mutant.

## Discussion

### Pleiotropic effects of the *pgp1-2* mutation on photosynthetic activities in the thylakoid membrane

In this study, the *pgp1-2* mutant, which lacks the ability to synthesize PG in plastids (Hagio et al., 2002), showed various defects in photochemical and electron transport activities in the thylakoid membrane. The very high Fo levels in the mutant (Figure 1D and Table 2) suggest that the energy transfer from antenna pigments to the open PSII reaction center is severely impaired, which results in loss of absorbed energy as fluorescence and decreased PSII photochemical efficiency. Dissociation of light-harvesting antenna complexes from the PSII core complex (Figure 8) may cause the energetic disconnection between the reaction center and antenna complexes in the mutant. Because high Fo is observed in mutants deficient in PSII (Meurer et al., 1996; Shikanai et al., 1999), PSII core components may be largely deficient in the *pgp1-2* mutant as suggested in the lack of oxygen evolution (Table 2). Upon illumination, chlorophyll fluorescence from the mutant PSII immediately reached nearly the maximal level (Figure 5), which reflects very small electron-accepting capacity of Q_A_ and the plastoquinone pool. Consistent with a larger decrease in reaction center proteins than that in LHC proteins in *pgp1-2* seedlings (Kobayashi et al., 2015), the mutant showed lower ratio of chl *a* to chl *b* and to carotenoids (Table 1), which may explain in part the decreased PSII capacity in *pgp1-2* plants. In addition, accumulation of oxidized P680, the primary electron donor of PSII, with the dysfunctional oxygen-evolving complex may inhibit repeated photochemical reactions in the mutant, thereby causing a rapid rise of chlorophyll fluorescence from the antenna systems. In the presence of DCMU, chlorophyll fluorescence from *pgp1-2* leaves was strongly quenched soon after peaking (Figure 5). Interestingly, similar kinetic profiles were observed in spinach monomeric PSII core complexes incubated with PG or SQDG in the absence of DCMU (Kansy et al., 2014). Excessive imbalance in the thylakoid lipid composition with more or less anionic lipids may strongly perturb the intrinsic PSII electron transport.

*In vitro* biochemical analysis demonstrated that degradation of phospholipids in the pea thylakoid membrane did not decrease PSI activity (Jordan et al., 1983). Moreover, in the *Synechocystis* 6803 *pgsA* mutant, decreased PSI activity was observed only after a longer period of PG deprivation (>2 weeks; Domonkos et al., 2004). These data suggest less importance for PG in the function of PSI. Indeed, compared to the crucial defects of the mutant PSII, PSI seemed relatively active in *pgp1-2* plants (Figure 7A). Considering the high structural stability of PSI, the interaction of PG molecules with PSI core proteins may be stronger than with PSII, so the effect of PG deprivation may be primarily observed in the PSII function. However, kinetic analysis of P700 oxidation showed that the cyclic electron pathway is dysfunctional in the *pgp1-2* mutant (Figure 7B). Moreover, the P700 oxidation kinetics in the presence of MV was slower in the mutant than the wild type, which may be attributed to an enhanced charge recombination within the PSI reaction center and/or inefficient energy transfer from the LHCI as suggested in the 77K chlorophyll fluorescence (Figure 8). X-ray crystallographic analyses revealed that PG molecules are structural components of the PSI complex in both cyanobacteria (*T. elongatus*; Jordan et al., 2001) and plants (*Pisum sativum*; Qin et al., 2015). Therefore, PG in the PSI complex, which may be resistant to phospholipase treatments (Jordan et al., 1983) and short-term PG deprivation (Domonkos et al., 2004), may also play an important role in the PSI activity.

In *pgp1-2*, the PGP2 isoform is functional in ER (Tanoue et al., 2014). Although total PG content is substantially decreased in the mutant (Hagio et al., 2002; Kobayashi et al., 2015), whether PGP2 can provide a significant amount of PG for chloroplasts remains undetermined. We previously reported that phosphate starvation further decreased total PG levels in *pgp1-2* mutant, which resulted in complete loss of Fv/Fm despite of accumulation of thylakoid membrane glycolipids (Kobayashi et al., 2015). Thus, PG produced by PGP2 may be partially transported into chloroplasts and support the marginal photosynthetic activity in *pgp1-2* plants, but further depletion of PG by phosphate starvation may completely abolish the PSII photochemical activity.

### The *pgp1-2* mutation caused strong photodamage

The impaired energy transfer from antenna pigments to the PSII reaction center and decreased electron-accepting capacity of PSII plastoquinones could increase non-photochemical dissipation of light energy within the antenna system. The very high Y_NO_ levels in *pgp1-2* mutant (Figure 2B) represent enhanced dissipation of absorbed light energy as a non-regulated form of fluorescence or heat, which indicates limited photoprotective capacity in the mutant. In fact, the non-photochemical quenching in the *pgp1-2* mutant was predominantly caused by photoinhibition of PSII (Figure 2C), which is consistent with the *pgp1-2* PSII being very susceptible to photodamage under red light irradiation (Figure 3).

Recent studies have proposed that photodamage to PSII occurs in two steps: first, the Mn cluster of oxygen-evolving complex is damaged by UV or blue light presumably via direct excitation of Mn, and second, the photochemical reaction center of PSII is inactivated by photosynthetically active light absorbed by chlorophyll (Tyystjärvi, 2008; Murata et al., 2012). Irradiation of red light alone has little effect on the PSII photoinhibition in the wild type, as was reported previously (Ohnishi et al., 2005; Sarvikas et al., 2006; Takahashi et al., 2010), but it strongly inactivated the PSII photochemical reaction in the *pgp1-2* mutant (Figure 3). As suggested by the dissociation of Mn^2+^ ions from protein systems in the mutant leaves (Figure 4) and lack of oxygen-evolving activity in the thylakoids from the mutant (Table 2), the Mn cluster in *pgp1-2* mutant may be dysfunctional, so the mutant PSII may be susceptible to red light. The finding in *Synechocystis* 6803 that PG is required for the stabilization of the Mn cluster in the oxygen-evolving complex (Sakurai et al., 2007) supports this hypothesis. Because the effects of the *pgp1-2* mutation on photosynthesis were pleiotropic, other defects such as impaired photochemical reactions or acceptor-side limitations might also be responsible for photoinhibition of the mutant PSII.

The severe defects in leaf development with reduced mesophyll cells in *pgp1-2* plants (Hagio et al., 2002; Kobayashi et al., 2015) may be explained in part by photodamage of developing chloroplasts. Indeed, the growth of *pgp1-2* seedlings was damaged under continuous 75 μmol photon m^−2^ s^−1^ light (Supplemental Figure 1). However, the development of *pgp1-2* leaves was perturbed even under dim light (< 5 μmol photon m^−2^ s^−1^), which suggests a specific role of PG synthesized in chloroplasts in leaf development.

### Effect of the *pgp1-2* mutation on the acceptor side of PSII

In *Synechocystis* 6803, deprivation of PG caused impaired electron transport from Q${}_{\text{A}}^{-}$ to Q_B_, and the impairment was further enhanced by BQ treatment (Gombos et al., 2002; Itoh et al., 2012). Retarded electron transfer at the acceptor side of PSII was also observed in the *pgp1-2* thylakoids (Figure 6A), consistent with the reports that phospholipase treatment inhibits electron transport at the Q_B_ site in thylakoid membranes isolated from plant leaves (Droppa et al., 1995; Kim et al., 2007). However, unlike in the *pgsA* mutant, in *pgp1-2* leaves, BQ treatment did not inhibit Q_A_ reoxidation in thylakoids. In *Synechocystis* 6803, PG deficiency may change the structure around the Q_B_ site so that it becomes inactivated by BQ (Itoh et al., 2012). Furthermore, with site-directed mutations in the *Synechocystis* 6803 D1 protein disrupting interactions with PG around the Q_A_ binding site, the Q_B_-related site was sensitive to BQ-mediated inactivation, which suggests PG molecules near the Q_A_ site are important for the function of the Q_B_ site (Endo et al., 2015). Therefore, the mode of interaction between PG and proteins in the PSII reaction center may differ between *A. thaliana* and *Synechocystis* 6803, although both organisms require PG to maintain the function of the acceptor side of PSII.

### Involvement of PG in organization of antenna-core complexes of photosystems

Biochemical approaches with phospholipases have revealed the importance of PG for the assembly of PSII-LHCII complexes. Phospholipase A_2_ treatments disassembled the spinach PSII dimer into monomers (Kruse et al., 2000), pea LHCII trimers into monomers (Nussberger et al., 1993), and *A. thaliana* PSII-LHCII complexes into PSII and LHCII monomers (Kim et al., 2007). Consistent with these findings, the *pgp1-2* thylakoids at 77K emitted chlorophyll fluorescence at ~680 nm (Figure 8), which suggests accumulation of free LHCII uncoupled from PSII. Furthermore, 77K fluorescence emission at ~723 nm suggests dissociation of LHCI from the PSI core, which may cause slower P700 oxidation in the presence of MV by far-red light in *pgp1-2* mutant than the wild type (Figure 7B). In the crystal structure of the pea PSI-LHCI complex, 3 PG molecules were identified in LHCI in addition to one PG molecule locating between LHCI and the PSI core (Qin et al., 2015). Thus, PG molecules in the complex may be involved in the association of PSI with LHCI.

Formation of the supramolecular networks of photosystems in grana thylakoids is self-organized and the balance between negative thylakoid surface charges and cations such as Mg^2+^ would play a crucial role (Kirchhoff et al., 2007). Low salt conditions cause an intermixing and randomization of the protein complex along with unstacking of the grana thylakoids, presumably by electrostatic repulsion of negative thylakoid surface charges. Such changes increase spillover of excitation energy from the PSII antenna system to the PSI (Figure 8; Kirchhoff et al., 2007). Incubation of the unstacked thylakoids with cations induces restacking of grana and rearrangement of PSII-LHCII supercomplexes, which leads to reduced energy spillover from PSII to PSI (Figure 8). As in wild-type thylakoids, *pgp1-2* thylakoids show an increase and decrease in the fluorescence emission from PSI in response to elimination and re-addition of cations, respectively. Thus, PG may not be needed for the self-organization of macromolecular protein networks formed in stacked grana thylakoids, although it is essential for the intrinsic assembly of antenna complexes with photosystem reaction centers. These results are consistent with the observation that enhanced biosynthesis of glycolipids including SQDG by phosphate starvation caused extensive stacking of the thylakoid membrane in the *pgp1-2* mutant but could not induce association of LHC antennas with PS core complexes (Kobayashi et al., 2015).

We previously reported that the amount of PS core proteins was decreased more strongly than that of LHC proteins in *pgp1-2* plants, although downregulation of genes encoding PS core proteins (*psaA* and *psbA*) was weaker than that of *LHC* genes (*LHCA4* and *LHCB6*; Kobayashi et al., 2015). Considering the importance of PG for the assembly of PS-LHC complexes, PG in chloroplasts may be required for stabilizing the core proteins during translation or post-translational assembly in complexes.

## Conclusion

Loss of plastidic PG biosynthesis by the *pgp1-2* mutation caused severely impaired electron transfer both in donor and acceptor sides of PSII and energetic uncoupling between PSII and LHCII, which would cause strong photodamage to PSII and presumably affected leaf development in part. Functionality of PSI was also affected by the mutation. By contrast, an 80% reduction in the PGP1 activity in *pgp1-1* mutant caused no remarkable defects in photochemical and electron transport activities, although it decreased chlorophyll content and mesophyll cell number (Xu et al., 2002; Yu and Benning, 2003). Thus, in the *pgp1-1* mutant, photosynthetic efficiency may be maintained by fine-tuning the quality and quantity of photosynthetic components to the reduced PG content and by increasing another anionic lipid, SQDG, to substitute for some functions of PG. However, in the *pgp1-2* mutant, the stress with critical loss of PG in chloroplasts might exceed the homeostatic capacity of plants and cause fatal damage to photosynthetic machinery directly and indirectly.

## Author contributions

HW directed the study. KK and HW designed the experiments. KK and KE performed the experiments and analyzed the data. KK wrote the manuscript.

### Conflict of interest statement

The authors declare that the research was conducted in the absence of any commercial or financial relationships that could be construed as a potential conflict of interest.
