# Supplementary material for: Multiple Impacts of Loss of Plastidic Phosphatidylglycerol Biosynthesis on Photosynthesis during Seedling Growth of Arabidopsis
Source: Front Plant Sci. 2016 Mar 21;7:336. doi: 10.3389/fpls.2016.00336 (PMC4800280; doi:10.3389/fpls.2016.00336)
Supplement: Supplementary file 1 [file Image1.PDF]

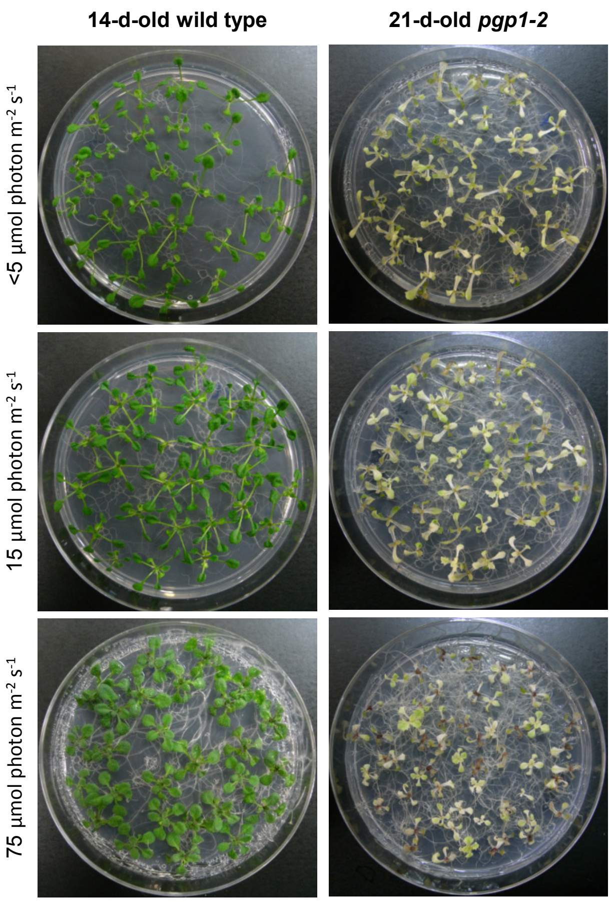

Supplemental Figure 1. Growth phenotypes of wild type and *pgp1-2* under different light intensities.
Wild-type and *pgp1-2* seedlings were grown under 15 µmol photon m^{-2} s^{-1} for 7 and 14 days, respectively, and were further grown under indicated light conditions for 7 days.
